# Evaluating essential medicines for treating childhood cancers: availability, price and affordability study in Ghana

**DOI:** 10.1186/s12885-021-08435-x

**Published:** 2021-06-10

**Authors:** Kofi Boamah Mensah, Adwoa Bemah Boamah Mensah, Varsha Bangalee, Neelaveni Padayachee, Frasia Oosthuizen

**Affiliations:** 1 Department of Pharmacy Practice, Faculty of Pharmacy & Pharmaceutical Science, College of Health Science, Kwame Nkrumah University of Science & Technology, Kumasi, Ghana; 2 Discipline of Pharmaceutical Sciences, College of Health Sciences, University of KwaZulu-Natal, Westville Campus, University Road, Durban, South Africa; 3 Department of Nursing, College of Health Science, Kwame Nkrumah University of Science & Technology, Kumasi, Ghana; 4 Department of Pharmacy and Pharmacology, University of the Witwatersrand, Johannesburg, South Africa

**Keywords:** Childhood cancer, Medicines, Availability, Price, Affordability, Ghana

## Abstract

**Introduction:**

Access to childhood cancer medicines is a critical global health challenge. There is a lack of sufficient context-specific data in Ghana on access to essential medicines for treating childhood cancers. Here, we present an analysis of essential cancer medicine availability, pricing, and affordability using the pediatric oncology unit of a tertiary hospital as the reference point.

**Method:**

Data on prices and availability of 20 strength-specific essential cancer medicines and eight non-cancer medicines were evaluated using the modified World Health Organization (WHO)/Health Action International method. Two pharmacies in the hospital and four private pharmacies around the hospital were surveyed. We assessed their median price ratio using the WHO international reference price guide. The number of days wages per the government daily wage salary was used to calculate the affordability of medicines.

**Results:**

The mean availability of essential cancer medicines and non-cancer medicines at the hospital pharmacies were 27 and 38% respectively, and 75 and 84% respectively for private pharmacies. The median price ratio of cancer medicines was 1.85, and non-cancer medicines was 3.75. The estimated cost of medicines for treating a 30 kg child with Acute lymphoblastic leukaemia was GHȻ 4928.04 (US$907.56) and GHȻ 4878.00 (US$902.62) for Retinoblastoma, requiring 417 and 413-days wages respectively for the lowest-paid unskilled worker in Ghana.

**Conclusion:**

The mean availability of cancer medicines at the public and private pharmacies were less than the WHO target of 80%. The median price ratio for cancer and non-cancer medicines was less than 4, yet the cost of medicines appears unaffordable in the local setting. A review of policies and the establishment of price control could improve availability and reduce medicines prices for the low-income population.

## Introduction

According to the World Health Organization (WHO) childhood cancer fact sheet, cancer is the primary cause of mortality in children and adolescents worldwide [1, 2]. Children living in high-income countries (HICs) constitute approximately 10% of the global children population where access to cancer care is optimal [3]. On the contrary, 90% of the global childhood cancers are recorded in low-and-middle-income countries (LMICs), yet, there is limited access to affordable medications in LMICs [4]. Consequently, survival estimates in LMICs are scandalously lower than those in HICs. As earlier indicated by the WHO, children with cancer in LMICs are four time more likely to die of the disease than children from HICs [5]. Reasons cited for this disproportional burden include late diagnosis of the disease, abandonment of treatment due to high cost and lack of specialized care [5].

In Ghana, available data indicate that the average number of new childhood cancers recorded at the two primary referral public hospitals, Korle-Bu Teaching Hospital and Komfo Anokye Teaching Hospital were 124 and 103 cases per year respectively [6, 7]. Studies suggest that the treatment of childhood cancers in LMICs is economically feasible [8, 9]. However, access to cost-effective healthcare, including essential cancer medicines, is a significant challenge [10]. In Ghana, childhood cancers are not integrated into national social programs. Hence, children with cancer do not benefit from health packages and their treatment are not covered under the National Health Insurance Scheme (NHIS) [11]. Thus, their healthcare services, including essential cancer medicines, are paid out-of-pocket. This phenomenon, as suggested by many studies, lead children with symptoms suggestive of cancer and their parents/guardians to seek healthcare from herbalist and sometimes at private and other lower-level health facilities instead of specialist cancer center. This consequently leads to delay in diagnosis and treatment [12, 13]. Therefore, it is essential to evaluate the cost, affordability and availability of childhood cancer medicines in Ghana to understand the caregivers’ burden and also inform policies.

In 2009, the WHO Expert Committee on the selection and use of Essential Medicines discussed the importance of including cancer medicines on the List of Essential Medicines for Children (EMLc) [14]. In 2011, the Expert Committee approved the inclusion of cancer medicines for the treatment of Wilms tumor, acute lymphoblastic leukemia (ALL), and Burkitt lymphoma in the EMLc (Table 1) [14]. The overall aim for the development of EMLc by the Expect Committee aimed to serve as guidance for LMICs to create their country-specific lists to improve the availability, affordability, and accessibility of essential cancer medicines required for the treatment of childhood cancers [10]. It further set a target of 80% availability of essential childhood cancer medicines [10] across the public and private health care sectors.

**Table 1:** Availability and price paid by clients at the hospital pharmacies and private pharmacies for surveyed medicines and their MPRs

	Availability			Price			
Medicine	Brand (IB/GB)	% Hospital Pharmacies (n = 2)	% Private Pharmacies (n = 4)	Median Price (Gh¢)	Minimum Price (Gh¢)	Maximum Price (Gh¢)	MPR
Dactinomycin, powder inj,500 μg in vial	GB	0	100	84	60	98.8	0.63
L-Asparaginase, powder inj, 10,000 IU in vial	GB	0	50	310.75	270	351.5	3.88
Carboplatin, inj, 150 mg/15 mL	GB	0	100	150	143	170	2.07
Carboplatin, inj, 450 mg/45 mL	GB	100	100	425	386	460	2.43
Etoposide, inj, 100 mg/5 mL	GB	100	100	40	35	52	2.12
Ifosphamide, powder inj, 1 g in vial	GB	0	50	155	140	170	3.36
Bleomycin, inj, 30 IU	GB	0	75	145	110	150	1.62
Cyclophosphamide, powder inj, 500 mg in vial	GB	100	100	25	17.63	35	0.72
Doxorubicin, powder inj, 50 mg in vial	GB	100	100	71	70	82	2.18
Vincristine, powder inj, 1 mg in vial	GB	0	100	27.5	20	35	1.54
6-Mecarptopurine, tab, 50 mg	GB	0	50	16	3	29	1.52
Dacarbazine, powder inj, 200 mg in vial	GB	0	50	79	55	104	1.15
5-Florouracil, inj, 500 mg/10 mL	GB	50	100	14	7.32	15	4.79
Methotrexate, inj, 50 mg in ViaL	GB	0	50	44	38	50	2.74
Methotrexate, tab, 2.5 mg	GB	0	50	4.9	3.8	6	13.19
Leucovorin,inj, 30 mg in vial	GB	0	50	87.5	75	100	1.25
Procarbazine, tab, 50 mg	GB	0	0	0	0	0	0
Chlormbucil, tab, 2 mg	GB	0	75	21	9.5	40	0.13
Cytarabine, inj, 100 mg in vial	GB	0	100	47.2	45	97	2.09
Granisetron, inj, 1 mg in vial	GB	100	100	28	20.67	68.83	0.8
**Mean (SD)**		**27(44)**	**75(29)**				
**Median MPR of GB**							**1.85**
**Non-cancer essential medicines**							
Dexamethasone, tab, 0.5 mg	GB	0	0	0.04	0.02	0.05	3.75
Dexamethasone, tab, 4 mg	GB	0	100	4	2	5	3.75
Dexamethasone, inj, 8 mg/2 ml	GB	50	100	1	0.8	1	0.65
Prednisolone, tab, 5 mg	GB	0	75	1.1	1	1.2	23.33
Hydrocortisone, inj, 100 mg in vial	GB	100	75	5	4	6	2.72
Allopurinol, tab, 100 mg	GB	50	75	0.72	0.56	1	51.43
Allopurinol, tab, 300 mg	GB	50	75	1.75	1.3	2	79.55
Nexium, powder, 10 mg	IB	50	100	6.85	3	10	0.51
**Mean (SD)**		**38(35)**	**84(13)**				
**Median MPR of GB**							**3.75**

IB, Innovator Brand; GB, Generic Brand; MPR, Median Price Ratio

One of the milestones in achieving the Sustainable Development Goals (SDGs), in particular SDG target 3.4 in LMICs is to reduce premature mortality from childhood cancers through the provision of cost-effective medicines. In September 2018, the WHO announced a new effort- the WHO Global Initiative for Childhood cancer – with the goal of reaching at least a 60% survival rate for children with cancer by 2030 [5]. The aim of this initiative is in two-fold [1]; increase prioritization of childhood cancer through awareness raising at global and national levels and [2] expand the capacity of countries in cancer diagnosis and treatment including availability of medicines and to deliver best practices [5]. The significant barrier to access essential cancer medicines in LMICs is high prices. If as a society, we are going to make positive development towards Target 3.4 to reduce premature mortality from childhood cancers and achieve 60% survival rates in less developed countries such as Ghana, we will have to develop policies that will improve the affordability, availability, and prices of childhood cancer medicines. Measuring and evaluating essential medicines prices are vital activities as these findings will inform the development of such policy and practice guidelines.

The WHO/Health Action International (HAI) has designed a method to determine the price, affordability, and availability of essential medicines [15]. The WHO/HAI method employs the Management Sciences for Health (MSH) international reference prices, which are globally accepted as the reference model, informing realistic procurement prices acquired through global tender prices and non-profit distributors to LMICs [16]. Considering the significance of essential cancer medicine prices and availability in patient access, WHO set a price target of not more than four times the international reference price across the public and private health care sectors [10].

The WHO/HAI method has been used to evaluate availability, price, and affordability of essential medicines in many regions of the world [10] but it has not been used to evaluate essential childhood cancer medicines in Ghana. The Ghana EMLc includes all the cancer medicines approved by the WHO Expert Committee, and it is vital to find out if these cancer medicines are easy-to-access by patients. Thus, the study aimed to access the availability, pricing, and affordability of essential cancer medicines in Ghana using the pediatric oncology unit of a tertiary hospital as the anchor point.

## Methods

A cross-sectional survey was undertaken using a modified form of the WHO/HAI method [17] to measure the availability, affordability, and price of childhood cancer medicines in the private and public retail pharmacies in the Ashanti region of Ghana. This region has one of the two national pediatric oncology centers for treating childhood cancer. Also, it is the most populated region in the country, with the highest incidence and prevalence rates of childhood cancers [6]. All methods used to generate data for this study were carried out in accordance with relevant guidelines and regulations of the Pharmacy Council of Ghana. Further, formal permissions were obtained from 4 private retail pharmacies and pharmacies of a tertiary health care facility involved in this study prior to the commencement of data collection (RD/CR20/155). For each of the 28 medicines included in the study, availability, and pricing data were collected for innovator brand and lowest price generic brand if available. The innovator brand is the first medicine created containing the exact active ingredient to receive approval for use. Whiles, the generic brand is a medicine created to be similar to an already marketed brand-name medicine in dosage form, strength, safety, route of administration, performance characteristics, quality, and intended use [18]. Ethical clearance for the study was granted by the Committee on Human Research, Publication and Ethics, Kwame Nkrumah University of Science and Technology, Ghana (Ref: CHRPE/AP/467/20).

## Sampling plan

### Survey facilities

There are two main tertiary hospitals in Ghana treating childhood cancers, each located in two different regions of the country. We purposively selected Komfo Anokye Teaching Hospital, located in the Ashanti region due to its geographical location, which is in the middle belt of Ghana, closer to the many regions and neighboring countries. The hospital is the closest teaching and referral center for childhood cancers for almost ten of the 16 regions of Ghana. This sampling method gives a maximum representative variation in determining the price and availability of essential medicines for childhood cancers in Ghana. The hospital was used as a ‘survey anchor”. The hospital has two pharmacies that stock cancer medicines. We surveyed the two-hospital pharmacies and four private pharmacies because they were the only pharmacies in the region which stock cancer medicines. Two of the four private pharmacies were closer to the survey anchor. These private pharmacies were well-known in the public arena to stock cancer medicines; hence data collectors easily located these facilities. The total sample size was six retail pharmacies.

### Survey medicines

The survey medicines were selected from the 2017 WHO EMLc model [19] and the WHO/HAI Global Core List [20]. The 2017 WHO EMLc suggests 28 cancer medicines and supportive care essential medicines for the treatment of childhood cancers. Also, WHO/HAI Global Core List contains 14 medicines chosen based upon the global burden of disease and also contained all WHO/HAI surveys. Twenty [20] dosage strength-specific essential cancer medicines and eight non-cancer essential medicines were selected. The selection was based on utilization, existing availability, and medicines used for the ten childhood cancers commonly diagnosed in Ghana (Burkitt’s lymphoma, Wilm’s tumour, acute lymphoblastic leukaemia, retinoblastoma, rhabdomyosarcoma, neuroblastoma, liver tumours, Ewing sarcoma, brain and spinal tumours, and germ cell tumours). All 28 survey medicines have lost their patent in major international markets. To ensure that the survey medicines are included in Ghana Essential Medicines List (EML) and approved for use in Ghana, the WHO EMLc and WHO/HAI Global Core List was compared with the Ghana EML. The eight commonly used non-cancer essential medicines at the paediatric oncology unit of the hospital served as a control or comparator to measure the difference in accessibility to both cancer medicines and other essential medicines.

The data collection sheet comprised a list of both innovator and generic brand essential cancer and non-cancer medicines with specific dosage form and strength. The innovator brand essential cancer and non-cancer medicines were identified at the survey site. Where necessary, an internet search and contacts with the pharmaceutical company’s local representative on the medicinal product were employed to verify the brand (innovator or generic) of the medicine.

## Data collection and analysis

An assessment was done to explore the viability of the study at the selected sites. This assessment included off-peak periods at the retail pharmacies, duty roaster of pharmacists at the sites, and restocking periods. Trained research assistants visited the selected retail pharmacies, engaged the pharmacists on duty on the survey day, and had them fill a data collection sheet designed by the authors. KBM and ABBM cross-checked the price and physical availability of the survey medicines at the sites.

### Availability

The availability of anti-cancer medicines was measured by physical inspection of the innovator or generic brand and dosage-strength of the medicines. The percentage availability of medicine at each survey site was calculated as:

$$ =\frac{\mathrm{Physical}\ \mathrm{availability}\ \mathrm{of}\ \mathrm{medicine}\ \mathrm{at}\ \mathrm{hospital}/\mathrm{private}\ \mathrm{pharmacies}}{\ \mathrm{Number}\ \mathrm{of}\ \mathrm{hospital}/\mathrm{private}\ \mathrm{pharmacies}\kern1.25em }\times 100 $$

If other than one dosage strength was listed on the WHO EMLc, WHO/HAI Global Core List and Ghana EML for a given a medicine (12 out of the 28 survey medicines), it was regarded available if only one of the dosage-strength was available at the survey site.

### Price

Ghana’s NHIS does not support anti-cancer medicines for the management of childhood cancers. All expenditures for the management of childhood cancer are paid out of pocket. Data on price were gathered from the public retail pharmacy (*n* = 2) and private retail pharmacy (*n* = 4) from their current price lists. The prices of both innovator and generic medicines that were available were recorded in Ghana cedis (GH¢). The price for injectables and solid dosage forms (tablet or capsule) were recorded per vial/ampoule and per tablet/capsule respectively. The median price ratio (MPR) was determined to evaluate the consumer price in Ghana compare to international reference prices. According to the WHO, no patient should pay for medicine, which is four times the international reference price [21]. The median price ratio (MPR) was calculated using the formula below:

$$ \mathrm{MPR}=\frac{\mathrm{Local}\ \mathrm{consumer}\ \mathrm{price}\ \left(\mathrm{USD}\right)}{\mathrm{International}\ \mathrm{reference}\ \mathrm{price}\ \left(\mathrm{USD}\right)}\ \mathrm{X}\ 100 $$

Medicines prices collected from the survey sites were for both generic and innovator brands if available. Supplementary information on procurement prices was obtained from the Medicines Management unit of the hospital. The private pharmacies did not provide additional information on procurement prices from their wholesalers because it was deemed as confidential.

### Affordability

The affordability was measured using two most common presenting childhood cancers at the anchor hospital, namely high risk-acute lymphoblastic leukemia and retinoblastoma. According to the WHO/HAI method, affordability is measured by calculating for the cost of a month supply of medicines for a treating a particular disease. In this study, affordability was calculated for the duration of therapy stated in the chemotherapy protocol (modified Red Cross Children Hospital Chemotherapy Protocol) use at the hospital. Thus, our affordability assessment used the minimum and maximum price of cancer medicine to calculate the cost of medicines required to treat a child with an average weight of 30 kg and a body surface area of 1m^2^, diagnosed of retinoblastoma or high risk-acute lymphoblastic leukemia. The number of days wages needed to pay for the total cost of therapy for each of the two-childhood cancer was obtained by; dividing the total cost of chemotherapy medicines required to treat each cancer by the daily minimum wage in Ghana. The daily minimum wage in Ghana is GhȻ 11.82 per day [22] and equivalent to 2.04 USD per day.

## Results

### Availability

The mean availability of cancer medicines at the survey sites was 27 and 75% for hospital and private pharmacies, respectively. Five medicines (Intravenous (I.V.) Carboplatin 450 mg, I.V. Etoposide 100 mg, I.V. Cyclophosphamide 500 mg, I.V. Doxorubicin 50 mg, and I.V. Granisetron 1 mg) from the WHO EMLc were available at all the survey site. The solid oral dosage form of procarbazine 50 mg was not available at all the survey site, and it was the only medicine which was not available at the private pharmacies. Fourteen cancer medicines from the survey list were not available at the hospital pharmacies. There were no innovator brand cancer medicines at all the survey sites.

The mean availability of non-cancer medicines at the hospital and private pharmacies was 38 and 84% respectively. Only I.V. Hydrocortisone 100 mg was available at all the hospital pharmacies. Only three medicines (Dexamethasone 4 mg tablet, I.V. Dexamethasone 8 mg and Nexium 10 mg powder) were available at all the private pharmacies. Nexium 10 mg powder was the only innovator brand non-cancer medicine that was available at the private pharmacies.

### Price

The MPR for generic brand cancer medicines and non-cancer medicines were 1.85 and 3.75, respectively. Two cancer medicines (Methotrexate tablet 2.5 mg and I.V. 5-fluorouracil 500 mg) recorded MPR of more than 4 times the international reference prices. Three non-cancer medicines (Prednisolone 5 mg tablet, Allopurinol 100 mg and 300 mg) recorded MPR of more than four times the international reference price. The MPR of the only innovator brand medicine (Nexium 10 mg powder) was less the 4. The lowest MPR was 0.13 for generic brand cancer medicine (Chlorambucil 2 mg tablet), and the highest MPR was 13.19 for generic brand cancer medicine (Methotrexate 2.5 mg tablet). The lowest recorded MPR for non-cancer medicine (Nexium 10 mg powder) was 0.51 and 79.55 for the highest recorded non-cancer medicine (Allopurinol 300 mg tablet). Table 1 presents the details of medicines availability and cost.

### Affordability

The total cost of medicines to treat a child with an average weight of 30 kg, and a body surface area of 1m^2^ diagnosed of high risk acute lymphoblastic leukemia (ALL)was GhȻ 4928.04 (US$907.56), when generic cancer medicines with the minimum retail price was used. However, the cost was GhȻ 10,929.3 9 (US$ 2012.76) when the maximum retail price of generic medicines was used. A minimum-waged unskilled government employee in Ghana earns GhȻ 11.82 per day and will have to work for 417 days to be able to afford the lowest-priced generic medicine for the treatment of ALL. For the worker to afford the maximum priced generic medicines as aforementioned, he/she will have to work for 925 days. In the instance of a diagnosis of retinoblastoma, the total cost of treatment will be GhȻ 4878 (US$ 902.62) using the minimum priced generic medicines and GhȻ 6474 (US$1197.94) using the maximum priced generic medicines. A minimum-waged unskilled government employee in Ghana will have to work for 413 days and 548 days to be able to afford the minimum and maximum price generic medicines respectively for the treatment of retinoblastoma. Table 2 presents details of the affordability assessment.

**Table 2:** Cost and affordability of medicines required to treat a 30 kg child with high risk acute lymphoblastic leukaemia and retinoblastoma

Disease	Duration of chemotherapy (weeks)	Total medicine cost using minimum GB prices (GhȻ)	Total medicine cost using minimum GB prices (US$)	Days wages needed to pay for total medicines for treatment	Total medicine cost using maximum GB prices (GhȻ)	Total medicine cost using maximum GB prices (US$)	Days wages needed to pay for total medicines for treatment
High-risk ALL	46	4928.04	907.56	417	10,929.30	2012.76	925
Retinoblastoma	15	4878.00	902.62	413	6474.00	1197.94	548

ALL, Acute lymphoblastic leukaemia; GB, Generic Brand

## Discussion

As far as it is known, this is the first comprehensive analysis of availability, prices, and affordability of paediatric cancer medicines in Ghana. The overall availability of cancer medicines fell below the WHO target for essential cancer medicines of 80% [23, 24]. The mean availability of these medicines was poor at the hospital pharmacies (27%) compared to the private pharmacies (75%). Chemotherapy service is provided at the hospital, so ideally the availability of cancer medicines is expected to be high at the hospital. The unsatisfactory availability of cancer medicines at the hospital pharmacies may indicate insufficient government funding for childhood cancer medicines. This is evidenced by the government 2019 budget to the Ghana Health Service which consisted of 99.8% for employee’s emolument and only 0.2% for services and goods [25]. The Internally Generated Fund (IGF) has been used as the primary source of funding for services and goods in the health sector. Nevertheless, IGF is meant to be spent on clinical services only [25]. This indicates that there are no or little funds provided to pay for medicines at even the basic level of the health system [25]. Simpler studies done in low-middle-income country’s (LMICs) such as Dar es Salaam (Tanzania) and Punjab (Pakistan) also reported poor availability of cancer medicines at public or government hospital pharmacy than the private pharmacy [26, 27]. The study done in Dar es Salaam concluded that government budget allocated for the purchase of cancer medicines was very small compared to the rising cases of both childhood and adult cancers. The Pakistan study also indicated that the government of Pakistan cannot maintain good public healthcare because of financial limitations [28]. Hence, government hospitals mostly experience a shortage of medicine [26].

Other factors contributing to inadequate availability of cancer medicines at the hospital pharmacies may be due to delays in awarding tenders, long lead times, failure to pay suppliers, poor supplier performance, and failure of suppliers to meet demands. Some of these contributing factors have been reported in other LMICs like Kenya and South Africa [29, 30].

The broader availability of cancer medicines at the private pharmacies may be explained by the high productivity and maximization of profit of the private sector, as seen in a market-oriented economy like Ghana.

The availability of five medicines (I.V. Carboplatin 450 mg, I.V. Etoposide 100 mg, I.V. Cyclophosphamide 500 mg, I.V. Doxorubicin 50 mg, and I.V. Granisetron 1 mg) at all survey site may be due its high demand because of its indication in the five common childhood cancers and other various cancers.

Our study highlights little significance in assessing or exploring the availability of innovator brand cancer medicines as shown by other studies [10, 31, 32]. Most innovator brand cancer medicines have lost their patent protection; hence, generic brands are widely available than innovator brands [33], and substitution with a generic brand is widely recognized in the public and private health system. .

The median MPR of all surveyed medicines, both cancer, and non-cancer medicines, were less than four, demonstrating comparatively lower local prices to international reference prices. Most of the generic cancer medicines available in Ghana are imported from India. This is evident in data from TradeMap, which suggests that $282 million value of pharmaceuticals was imported into Ghana in 2018, out of which 65% were from India [34]. This is because India has a large number of generic manufacturers [35–37] and also has the lowest consumer prices on cancer medicines [38]. Hence, this may explain the low median MPR achieved in the survey.

In Ghana, there are no regulatory or legal provisions controlling the pricing of medicines .The government does not provide an active nationwide medicines price regulation system for retail prices [39]. There is no existence of any legislation that mandates that medicine price information should be publicly available [40]. This may explain why some medicines (oral Methotrexate and I.V. 5-fluororuracil, Prednisolone 5 mg, Alopurinol 100 mg and 300 mg) were more than four times the international reference price. The authors recognize that there may be diverse economic drivers that can determine medicine prices but are not considered in this study.

The affordability of medicine is considered reasonable if the cost of treating a disease course is equal to or less than a day wage of the minimum-paid government employee [41]. Nevertheless, cancer treatment required more than one working day to pay for a day’s treatment, which is considered unaffordable to most patients [26, 27]. The price of the surveyed medicines was comparatively low to international reference prices. However, the cost of chemotherapy treatment for ALL and retinoblastoma was not affordable. Our result is similar to other studies done in LMICs, which reported that cancer medicines are not affordable [26, 27, 42].

### Strengths and limitations

This is the first survey on the availability, prices, and affordability of essential cancer medicines using the WHO/HAI methodology. This survey gathered data from pharmacies in one of the two public cancer referral hospitals and private pharmacies specializing in stocking and marketing of cancer medicines. Future research should focus on assessing the availability, prices, and affordability of cancer medicines in the other public cancer referral hospital in another region of Ghana. Our study had other limitations. Some medicines were reported on the day of data collection at the survey sites as ‘out-of-stock’. This may underestimate the availability of medicines at both the hospitals and private pharmacies since they may be re-stocked after the study period. Only one innovator brand was recorded to be available during the study, which made us deviate from the WHO/HAI methodology. However, this did not influence the results of the availability and affordability in general. Also, the affordability may have been underestimated as other service costs, and non-medical costs (hospital admission or laboratory tests) were not included in the treatment of various cancers. The study was a cross-sectional design hence fluctuation or pattern in medicine availability and price overtime was not captured. The study findings cannot generalize for the country, as this was conducted in one geographical region of Ghana. Other limitations of the methodology are the small number of cancer medicines surveyed and not considering all price parts in the pharmaceutical supply chain.

## Conclusion

Most anti-cancer medicines surveyed were found in the private pharmacy; however, the mean availability across all surveyed pharmacies was below the WHO set target of 80%. The low availability of medicines at the public pharmacies indicates the need for government intervention and strengthening of the existing procurement, supply, and distribution system at the hospital.

The price of surveyed medicines was below the international reference price but unaffordable for the government minimum-paid worker in Ghana. Children with cancer require a reliable supply of affordable cancer medicines. The absence of such a supply of affordable medicines can lead to preventable mortality and morbidity.

A range of policies and technical options are available for the government to ensure that cancer medicines are available and affordable. We recommend some of these options such as improvement in price transparency, health insurance schemes for childhood cancers, subsidies for essential childhood cancer medicines, prioritizing cancer medicines for national EMLc, and importation of cancer medicines by the government.

This work supports the international agenda aimed to improve global childhood cancer outcomes [43] by providing baseline data on access to essential childhood cancer medicines in Ghana.

## Data Availability

All data generated or analyzed during this study have been deposited into Figshare and can be access at https://figshare.com/s/a24fefdb416131aa4408.

## Abbreviations

WHO: World Health Organization
HAI: Health Action International
LMICs: Low-middle-income countries
HICs: High income countries
NHIS: National Health Insurance Scheme
EMLc: Essential Medicines List for children
SDGs: Sustainable Development Goals
MSH: Management Science for Health
I.V.: Intravenous
MPR: Median Price Ratio
ALL: Acute Lymphoblastic leukemia
USD: United State Dollar

## References

[1] Steliarova-Foucher E, Colombet M, Ries LA, Moreno F, Dolya A, Bray F (2017). International incidence of childhood cancer, 2001–10: a population-based registry study. The Lancet Oncology.

[2] The Global Cancer Observatory. GHANA SOURCE, GLOBOCAN, 2018: 2019; 2019 [cited 2020 07/2020]. Available from: https://gco.iarc.fr/today/data/factsheets/populations/288-ghana-fact-sheets.pdf.

[3] Rodriguez-Galindo C, Friedrich P, Alcasabas P, Antillon F, Banavali S, Castillo L, Israels T, Jeha S, Harif M, Sullivan MJ, Quah TC, Patte C, Pui CH, Barr R, Gross T (2015). Toward the cure of all children with cancer through collaborative efforts: pediatric oncology as a global challenge. J Clin Oncol.

[4] Bhakta N, Force LM, Allemani C, Atun R, Bray F, Coleman MP, Steliarova-Foucher E, Frazier AL, Robison LL, Rodriguez-Galindo C, Fitzmaurice C (2019). Childhood cancer burden: a review of global estimates. The lancet oncology.

[5] World Health Organization. Global Initiative for Childhood Cancer Geneva: World Health Organization; 2018 [updated 04/10/2018; cited 2020 07/2020]. Available from: https://www.who.int/news-room/detail/04-10-2018-global-initiative-for-childhood-cancer.

[6] Paintsil V, Dogbe J, Blay N, Osei Akoto A, Osei Tutu L, Hammond C. Pattern of Childhood Cancers Presenting to the Paediatric Cancer Unit of a Tertiary Hospital in Kumasi, Ghana. 2015.

[7] Segbefia C, Renner L, Dei-Adomakoh Y, Welbeck J (2013). Changing pattern of childhood cancers at Korle Bu teaching hospital, Accra, Ghana. Postgrad Med J Ghana.

[8] Bhakta N, Martiniuk AL, Gupta S, Howard SC (2013). The cost effectiveness of treating paediatric cancer in low-income and middle-income countries: a case-study approach using acute lymphocytic leukaemia in Brazil and Burkitt lymphoma in Malawi. Arch Dis Child.

[9] Russell HV, Panchal J, VonVille H, Franzini L, Swint JM (2013). Economic evaluation of pediatric cancer treatment: a systematic literature review. Pediatrics..

[10] Faruqui N, Martiniuk A, Sharma A, Sharma C, Rathore B, Arora RS, Joshi R (2019). Evaluating access to essential medicines for treating childhood cancers: a medicines availability, price and affordability study in New Delhi, India. BMJ Glob Health.

[11] Graphic Online. Put childhood cancers on NHIS. 2019.

[12] Chakrabarty J, Pai MS, Ranjith V, Fernandes D (2017). Economic burden of cancer in India. Indian Journal of Public Health Research & Development.

[13] Mohanti BK (2011). Estimating the economic burden of cancer at a tertiary public hospital: a study at the all India Institute of Medical Sciences.

[14] Robertson J, Magrini N, Barr R, Forte G, Ondari C (2015). Medicines for cancers in children: the WHO model for selection of essential medicines. Pediatr Blood Cancer.

[15] World Health Organization. Measuring medicine prices, availability, affordability and price components. World Health Organization; 2008.

[16] Management Sciences for Health. International medical products price guide USA; 2016.

[17] Raju PKS. Chapter 6.2 - WHO/HAI Methodology for Measuring Medicine Prices, Availability and Affordability, and Price Components. In: Vogler S, editor. Medicine Price Surveys, Analyses and Comparisons: Academic Press; 2019. p. 209–28.

[18] US Food & Drug Administration. Generic Drug Facts United States of America: US Food & Drug Administration,; 2018 [cited 2020 07/2020]. Available from: https://www.fda.gov/drugs/generic-drugs/generic-drug-facts#:~:text=A%20generic%20drug%20is%20a,%2C%20quality%2C%20and%20performance%20characteristics.

[19] World Health Organization. The selection and use of essential medicines: report of the WHO Expert Committee, 2017 (including the 20th WHO Model List of Essential Medicines and the 6th Model List of Essential Medicines for Children): World Health Organization; 2017.

[20] Kasonde L, Tordrup D, Naheed A, Zeng W, Ahmed S (2019). Evaluating medicine prices, availability and affordability in Bangladesh using World Health Organisation and health action international methodology. BMC Health Serv Res.

[21] Sharma A, Rorden L, Ewen M, Laing R (2016). Evaluating availability and price of essential medicines in Boston area (Massachusetts, USA) using WHO/HAI methodology. Journal of pharmaceutical policy and practice.

[22] Mark-Anthony Vinorkor. National Daily Minimum Wage increased to GH¢11.82. Graphic Online. 2019.

[23] Cherny NI, Sullivan R, Torode J, Saar M, Eniu A (2017). ESMO international consortium study on the availability, out-of-pocket costs and accessibility of antineoplastic medicines in countries outside of Europe. Ann Oncol.

[24] Servan-Mori E, Heredia-Pi I, Montañez-Hernandez J, Avila-Burgos L, Wirtz VJ (2015). Access to medicines by Seguro popular beneficiaries: pending tasks towards universal health coverage. PLoS One.

[25] UNICEF Ghana. Health budget brief. Accra. 2019;2019.

[26] Sarwar MR, Iftikhar S, Saqib A (2018). Availability of anticancer medicines in public and private sectors, and their affordability by low, middle and high-income class patients in Pakistan. BMC Cancer.

[27] Yohana E, Kamuhabwa A, Mujinja P (2011). Availability and affordability of anticancer medicines at the ocean road Cancer Institute in Dar es salaam, Tanzania. East African journal of public health.

[28] Irfan SM, Ijaz A, Shahbaz S (2011). An assessment of service quality of private hospitals in Pakistan: a patient perspective. Indian Journal of Commerce and Management Studies.

[29] Modisakeng C, Matlala M, Godman B, Meyer JC. Medicine shortages and challenges with the procurement process among public sector hospitals in South Africa; findings and implications. BMC health services research. 2020;20(1):234-. 10.1186/s12913-020-05080-1 PMC7082963 32192481

[30] Muhia J, Waithera L, Songole R (2017). Factors affecting the procurement of pharmaceutical drugs: a case study of Narok County referral hospital. Kenya Med Clin Rev.

[31] Jiang M, Yang S, Yan K, Liu J, Zhao J, Fang Y (2013). Measuring access to medicines: a survey of prices, availability and affordability in Shaanxi province of China. PLoS One.

[32] Xi X, Li W, Li J, Zhu X, Fu C, Wei X (2015). A survey of the availability, prices and affordability of essential medicines in Jiangsu Province, China. BMC Health Serv Res.

[33] Kotwani A (2013). Where are we now: assessing the price, availability and affordability of essential medicines in Delhi as India plans free medicine for all. BMC Health Serv Res.

[34] Asokolnsight. Ghana's Leading Pharmaceutical Manufacturers Ghana: AsokoInsight; 2019 [updated 19/06/2019; cited 2020 07/2020]. Available from: https://asokoinsight.com/content/market-insights/ghana-leading-pharmaceutical-manufacturers.

[35] Cameron A, Ewen M, Ross-Degnan D, Ball D, Laing R (2009). Medicine prices, availability, and affordability in 36 developing and middle-income countries: a secondary analysis. Lancet.

[36] Dhamija P, Sharma PK, Kalra S (2015). Only generics (drugs/names): is India ready?. Indian journal of endocrinology and metabolism.

[37] Kishore SP, Basu S, Selvaraj S (2013). Access to cancer medicines in India. Lancet Oncol.

[38] Goldstein DA, Clark J, Tu Y, Zhang J, Fang F, Goldstein R, Stemmer SM, Rosenbaum E (2017). A global comparison of the cost of patented cancer drugs in relation to global differences in wealth. Oncotarget..

[39] Ministry of Health. Ghana National Drug Policy, Ghana2004 [Available from: http://apps.who.int/medicinedocs/documents/s16185e/s16185e.pdf.

[40] Medicines Transparency Alliance MeTA. Baseline Assessments Pharmaceutical Sector Scan I 2009 [cited 2020 07/2020]. Available from: http://www.medicinestransparency.org/fileadmin/uploads/Documents/countries/Pharma_Scan/MeTA_Ghana_Pharmaceutical_Sector_Scan_-_Summary_report.pdf.

[41] Khuluza F, Haefele-Abah C (2019). The availability, prices and affordability of essential medicines in Malawi: a cross-sectional study. PLoS One.

[42] Moye-Holz D, Ewen M, Dreser A, Bautista-Arredondo S, Soria-Saucedo R, van Dijk JP, Reijneveld SA, Hogerzeil HV (2020). Availability, prices, and affordability of selected essential cancer medicines in a middle-income country – the case of Mexico. BMC Health Serv Res.

[43] Tang B, Bodkyn C, Gupta S, Denburg A (2020). Access to WHO essential medicines for childhood Cancer Care in Trinidad and Tobago: a health system analysis of barriers and enablers. JCO Global Oncology.

